# Review Article: Overview of Clinical Genetics of Diabetes Mellitus

**DOI:** 10.3390/genes17020215

**Published:** 2026-02-10

**Authors:** Alexander Asamoah, Rexford S. Ahima

**Affiliations:** 1 Norton Children’s Medical Group Genetics Division, University of Louisville, Louisville, KY 40202, USA; 2 Department of Medicine, Division of Endocrinology, Diabetes, and Metabolism, Johns Hopkins University, Baltimore, MD 21218, USA; ahima@jhmi.edu

**Keywords:** diabetes mellitus, polygenic risk score (PRS), genome-wide association studies (GWAS), heteroplasmy, monogenic diabetes, penetrance, variable expressivity

## Abstract

**Background:** Diabetes mellitus is characterized by elevated blood sugar due to absolute or relative insulin deficiency. Diabetes is classified as type 1 (T1D) or type 2 diabetes (T2D), gestational diabetes, and other types, such as monogenic diabetes, exocrine pancreatic disorders, and medication-induced diabetes. **Objectives:** This review article provides an overview of diabetes genetics, covering polygenic, monogenic, and syndromic forms of the disorder with emphasis on aspects to help clinicians in diagnosis, management, and counseling, but also to foster valuable knowledge for diabetic researchers in identifying phenotypes that will help inform gene discovery. **Key Findings:** Most cases of T1D and T2D are polygenic with environmental triggers. T1D results from autoimmune destruction of pancreatic beta cells leading to absolute insulin deficiency. Genetic studies of T1D have focused on the identification of loci associated with increased susceptibility to T1D. Early studies showed a linkage between T1D and several human leukocyte antigen (HLA) susceptibility loci on chromosome 6. Genome-wide association studies (GWAS) have identified more than 100 HLA- and non-HLA loci that increase susceptibility to T1D. It has been well established that a substantial portion of the genetic risk for T1D is encoded in the HLA locus. The non-HLA loci *INS*, *CTLA4*, *IL2RA*, *IFIH1*, and *PTPN22* make moderate contributions to T1D risk. Many other non-HLA loci have small effects to the phenotype and are relevant to autoimmunity, but they are yet to be identified. T2D, on the other hand, is associated with obesity and insulin resistance with relative insulin deficiency. Thousands of gene variants that are common and contribute small effects have also been identified through GWAS to contribute to T2D risk, but the rarer variants may confer significant risk to an individual’s risk. Common variants in the *TCF7L2* locus consistently carry one of the largest risks associated with T2D with a reported 1.7-fold disease odds for homozygous carriers. The usefulness of individual variants for genetic counseling in the common forms of diabetes has been limited in clinical settings in the past. The development of polygenic risk scores (PRS) and partitioned polygenic risk scores (PPRS), statistics derived from GWAS, are being used to predict and classify diabetes. The performance of PRS and PPRS varies by ancestry and type of diabetes. The PRS performs better with T1D, with an area under the curve and receiver operating characteristics (AUC-ROC) ranging from 0.87 to 0.93, compared to 0.72–0.75 for T2D. The genetic architecture of T2D is markedly more polygenic than T1D, and the PPRS has been useful in assessing risk in that setting. Monogenic diabetes comprises several dysglycemic disorders that include neonatal diabetes, maturity-onset diabetes of the young (MODY), and other genetic syndromes that have diabetes either as an associated finding and/or as a complication. Some of the monogenic diabetes gene variants have incomplete penetrance and variable expressivity leading to different ages of onset and variable presentation even within the same family. Hence some patients with these conditions have been previously diagnosed as having T1D or T2D. Many monogenic disorders follow Mendelian inheritance patterns, so genetic counseling is relatively straightforward if pathogenic variants are found to be inherited from a parent. Counseling for forms of diabetes due to maternally inherited mitochondrial cytopathies, such as MELAS and Kearns–Sayres syndrome, is not straightforward due to the occurrence of two or more populations of genetically distinct mitochondrial DNAs in the cells (heteroplasmy); the higher the percent of pathogenic variants in a cell or tissue, the greater the chance for affectation of disorder. **Implications:** Early stages of diabetes may be asymptomatic, and improvement in methodologies to identify individuals at high risk is important so prevention strategies can be targeted to susceptible individuals to slow or obviate the onset of disease and to minimize complications. **Conclusions:** Diabetes is a heterogeneous disorder, and accurate definition of phenotypes in the setting of non-syndromic and syndromic forms, development of powerful statistical methodologies, use of next-generation sequencing applications to interrogate the genome, incorporation of epigenetic mechanisms in statistical modeling and accurate curation of gene variants, will help us to realize application of genomic medicine and to inform diabetes care.

## 1. Introduction

Diabetes mellitus is a clinical condition characterized by chronic hyperglycemia due to either absolute or relative insulin deficiency [1,2,3]. Diabetes is associated with a high morbidity and mortality from end-organ damage [1]. The American Diabetes Association Expert Panel defines diabetes in non-pregnant individuals as hemoglobin A1C greater or equal to 6.5%, or fasting plasma glucose level greater than or equal to 126 mg/dL (7.0 mmol/L), or a 2-h fasting plasma glucose level during oral glucose tolerance testing (OGTT) greater or equal to of 200 mg/dL (11.1 mmol/L), or a random fasting plasma glucose level greater or equal to 200 mg/dL (11.1 mmol/L) in an individual with symptoms of polyuria, polydipsia, or polyphagia with weight loss [2]. Table 1 shows the diagnostic algorithm for classic diabetes and impaired glucose tolerance (IGT).

Diabetes occurs worldwide. The World Health Organization reports that about 830 million people have the disease, with most affected persons living in low- and middle-income countries [3]. In 2022, it was estimated that about 14% of adults aged 18 and above were living with diabetes, which is double the 1990 rate [4]. The International Federation of Diabetes Atlas (2025) reports that 1 in 9 adults aged 20 to 79 have diabetes, with significant estimated increases in sub-Saharan Africa, the Middle East and North Africa, Southeast Asia, and South and Central America [5]. In the USA, it was estimated in 2021 that 97.6 million adults aged 18 years and older had prediabetes, and 1.2 million are diagnosed with diabetes every year [4]. Thus, diabetes is a major healthcare burden in all countries [4].

Diabetes mellitus is a genetically heterogeneous group of disorders [1,6,7,8,9]. Diabetes is classified into different categories, of which type 1 diabetes (T1D) and type 2 diabetes (T2D) are the most common. Type 1 diabetes (T1D) results from autoimmune destruction of pancreatic beta cells leading to absolute insulin deficiency [1,2]. Type 2 diabetes (T2D) is associated with insulin resistance and failure of pancreatic beta cells to produce enough insulin [1,6,7,9]. The risk of T2D increases with obesity, but it may occur in normal weight individuals [10]. Relative insulin insufficiency in T2D is attributed to an intrinsic insulin secretory defect and metabolic stress caused by glucotoxicity and lipotoxicity [11,12].

T1D and T2D are described as complex disorders in that different environmental factors interact with some genetic factors to cause the disorders. Some of the environmental factors include diet, obesity, infections, and physical inactivity [1,13]. T1D and T2D are heritable conditions with a heritability estimated to be over 50% [1]. Evidence for the high heritability is based on the finding of familial aggregation and twin studies largely in populations of European ancestry [1,14,15,16,17]. Decades ago, diabetes mellitus used to be regarded as the “geneticist’s nightmare” since elucidating the genetic etiologies was difficult to unravel. Genetic analysis was hampered by differences in the diagnosis of affected individuals, age of onset, high prevalence of the disease in the population, and gene–environment interaction [1,15,18]. Progress in gene mapping techniques and statistical genetics methodologies have helped in our understanding of risk assessment for affected family members to some extent. The methodologies used have included candidate gene approaches that utilized sib-pair linkage analysis and transmission disequilibrium tests, among others [1,15,18,19,20,21,22]. Hypothesis-free testing using genome-wide association studies (GWAS) has enabled interrogation of the genome using single nucleotide variants (SNVs) [22,23,24].

A polygenic risk score (PRS) is a statistical estimate of an individual’s genetic susceptibility to developing a complex disorder. A PRS has been used to study disorders such as diabetes, schizophrenia, bipolar disorder, ADHD, and Parkinson disease, among other disorders [25,26]. A PRS has also been used for normally distributed traits such as height. The PRS explores the genetic contribution to the etiology and prediction of individual disease risk or trait outcome. It is calculated by considering the cumulative effects of multiple genetic variants (SNVs) identified through GWAS that have been associated or linked to the disease in large population studies. The most common metric used to evaluate PRS performance is the area under the curve of the receiver operating characteristics (AUC-ROC). The AUC-ROC measures the ability to discriminate between cases and controls, balancing sensitivity (true positive rates) against specificity (true negative rates). An AUC-ROC of 0.5 indicates a predictor with no better than random predictive utility, and values close to 1.0 indicate ideal discrimination between cases and controls. A higher PRS indicates a higher genetic susceptibility to a disease. A PRS has been calculated for different populations, and it does not fully account for the complex interaction between genetic and environmental factors [25,26].

Partitioned polygenic risk scores (PPRS) provide estimates about the extent to which different pathophysiologic processes contribute to risk and provide insight into underlying molecular heterogeneity of the disorder [26]. The PRS and PPRS have been providing new insights into tissue and cell-specific pathways in our understanding of the etiology of diabetes mellitus [26,27,28]. GWAS conducted on thousands of subjects with T1D and T2D have been able to overcome clinical heterogeneity and have yielded a large number of genetic susceptibility variants for T1D and T2D [26,27,29,30]. The PRS and PPRS can identify individuals with a higher risk of T1D and T2D than those in the general population. Polygenic risk scores can also discriminate T1D from T2D and monogenic diabetes [30].

The original PRS and PPRS were developed using European GWAS data, but multi-ancestry models have been developed [26,28,31]. Some of the major obstacles to clinical utility of a PRS for T1D and T2D include their inconsistent performance in populations that have considerable ancestry-related heterogeneity. The PRS calculated for African cohorts with T2D tend to show substantial decreases when European-generated models are used, pointing to the need for ancestry-related models. However, difficulties with participant recruitment from these multi-ancestry populations exist due to resource allocation, and this affects the sample sizes required to achieve significant levels in PRS and PPRS modeling [26]. Currently, hundreds of HLA and non-HLA variants for T1D and thousands of variants for T2D are utilized in diabetes risk analyses. Continued investment in global consortia, such as the Type 1 Diabetic Genetics Consortium (T1DGC), the National Institute of Diabetes and Kidney Diseases (NIDDK), and the Wellcome Trust Case Control Consortium (WTCCC), for genetic variant mapping will help identify the genes involved in the pathophysiologic pathways involved in insulin secretion and signaling, and provide insight into new targets for the prediction, prevention, and treatment of diabetes. High-resource countries may have to invest more to support program developments among low-resource populations to address PRS and PPRS performance problems and to avoid reinforcing the already burgeoning healthcare disparities.

Other causes of diabetes mellitus include single gene defects (monogenic diabetes), pregnancy-induced (gestational) diabetes, diseases of the exocrine pancreas, endocrinopathies (Cushing syndrome, polycystic ovarian syndrome, acromegaly, and hyperthyroidism), surgical excision of pancreas, trauma, drugs or environmental chemical exposures, infections, metabolic disorders, and rare genetic syndromes [1,8].

Monogenic diabetes comprises several clinical dysglycemic disorders that include neonatal diabetes, maturity-onset diabetes of the young (MODY), and several genetic syndromes that have diabetes either as an associated finding and/or as a complication [32]. Some patients diagnosed with T1D or T2D may in fact have monogenic diabetes. The monogenic diabetes genes may have incomplete penetrance and variable expressivity leading to different ages of onset and variable presentation even within the same family [1,32,33]. Monogenic diabetes and diabetes associated with genetic syndromes tend to follow Mendelian inheritance patterns, so family counseling and risk assessment may be straightforward if causal pathogenic variants are found to be inherited from a parent [1,32,33]. Family risk assessment and counseling for de novo variants can be challenging since one cannot rule out the possibility of germline mosaicism. Counseling for forms of diabetes due to maternally inherited mitochondrial cytopathies are not straightforward because of heteroplasmy.

This review describes the clinical genetics of diabetes mellitus and discusses current genetic counseling strategies for polygenic, Mendelian forms, and other forms of diabetes mellitus that may have genetic underpinnings.

## 2. Type 1 Diabetes (T1D)

Type 1 diabetes is an autoimmune disorder culminating in the progressive destruction of pancreatic islet beta cells leading to profound insulin deficiency. T1D accounts for about 5% of diabetes. Incidence of T1D varies around the world, and has been increasing in the majority of the countries studied. The reason for the increase is unclear, but it is believed changing environmental factors may play a role. The age of onset of T1D is from childhood through adulthood and the symptoms include polyuria, polydipsia, polyphagia with weight loss, hyperglycemia, and ketosis. Classic symptoms occur after destruction of 80% to 90% of insulin-secreting islet cells [8,29]. The stages are now recognized in the onset of T1D: stage 1 represents the development of autoantibodies with normal blood glucose; stage 2 reflects the progression to asymptomatic dysglycemia; stage 3 represents the clinically overt disease requiring insulin therapy [5]. Testing for glutamic acid decarboxylase autoantibody (GAD), islet cell cytoplasmic antibody (ICA), isulinoma-associated-2 autoantibody (IA-2), insulin autoantibody (IAA), and Zinc transporter 8 autoantibody (ZnT8) help with diagnosis and prediction [8,29]. A positive test for two or more of these autoantibodies significantly increases the risk of developing T1D and often correlates with a younger age of onset and more severe insulin deficiency. In addition, the presence of autoantibodies signifies ongoing autoimmune attack on insulin-producing beta cells even before symptoms appear [5]. There is heterogeneity of the clinical and immunological features of T1D based on age of onset. Childhood T1D is usually characterized by sudden onset and ketosis, and affected individuals tend to have HLA-DRB1*04-DQA1*0301-DQB1*0302 alleles and a high frequency of insulin and IA-2 autoantibodies [8,29]. Classic T1D in adults also presents with autoimmune destruction of beta cells, like in children and adolescents, is often rapidly progressive leading to insulin dependence shortly after diagnosis, and may lack the “honeymoon” phase seen in pediatric TID.

Latent autoimmune diabetes of adults (LADA) is described as new-onset diabetes after the age of 35 years in a patient with clinical features similar to T2D who tests positive for islet immunogenetic markers associated with T1D. LADA is a slowly progressive form of autoimmune diabetes, accounts for 2–12% of all cases of adult-onset diabetes depending on the population, and is managed with diet and therapeutic strategies aimed toward preserving residual insulin secretory capacity. LADA is initially non-insulin dependent but later requires insulin treatment. LADA is characterized by the presence of glutamic acid decarboxylase-65 (GAD65) autoantibodies and/or islet cell antibodies [8,34,35].

T1D is a polygenic disease, and genetic factors may account for about 35% of the susceptibility to the disorder based on concordance between monozygotic twins [1,13]. Other studies have suggested that about 50% of the risk of T1D is due to genetic factors [1,8]. Heritability of T1D is estimated to be about 50% to 80% [13,14,15]. Early evidence of the high heritability of T1D has come from familial aggregation and twin studies largely from European populations. The concordance rate of T1D among monozygotic (MZ) twins who share 100% of their genes in common is estimated to be 30% to 70%, and the risk is highest if the MZ twins are diagnosed at a younger age. Dizygotic twins, on the other hand, share on average 50% of their genes in common and their concordance rate is much lower, at 6% to 10% [1]. In addition, the risk for T1D among first degree relatives (5% for a sibling with another first degree relative in European populations) is higher than for unrelated individuals in the same general population (1 in 300) [13]. The closer a person is to the affected individual (proband), the greater the risk of developing T1D (first degree relatives like parents and siblings have higher risk), and this risk diminishes with distant relationships (second, third degree relatives, etc.) [1,13].

Environmental factors have been implicated in the pathogenesis of T1D. Viral infections of rubella, Coxsackie, mumps, enterovirus, Epstein–Barr virus, and recently SARS-CoV-2 have been linked to the development and progression of T1D [36]. In vitro experiments have demonstrated that viral infection directly affects the secretion of pro-inflammatory mediators leading to the destruction of islet beta cells. Other studies have suggested an indirect mechanism by which similarities between the amino acid sequence of the beta cell-specific GAD and proteins derived from viruses trigger the process of autoimmune destruction of beta cells [36]. Other environmental factors include early introduction to cow’s milk, vitamin D, and the gut microbiota that can affect the immune system [1,13].

Family linkage studies have demonstrated that alleles in the HLA region, which mediate immune function, impart a large increase in the odds of developing T1D. The HLA haplotypes DR3 (DQb1*201) and/or DR4 (DQb1*302) on chromosome 6 are susceptibility alleles, and DR2 (DQb1*602) is considered a protective allele for the development of T1D [1,19,20]. Up to 90% of individuals with T1D carry the HLA class II haplotype DR4-DQ8 and/or DR3-DQ2. Historically, T1D was thought primarily to affect peoples of European ancestry, but evidence suggests that the most significant increase in recent incidence is in non-European populations. Studies in populations of African, Japanese, and Afro-Caribbean ancestry have showed significant association with T1D and loci in the HLA region, but the specific alleles and their strength vary. For example, the DRB1*09-01 confers a high risk of T1D in Asians but not in Europeans, and the African-specific HLA-DR3 and HLA-DR7 have the opposite effects on T1D susceptibility in Europeans. In designing GWAS-associated PRS and PPRS studies, it is important for diabetes researchers to include non-European cohorts to improve the behavior of these statistical methods in risk assessment.

Genome-wide association studies (GWAS) have identified hundreds of HLA and non-HLA loci that increase susceptibility to T1D [29]. Apart from the HLA locus, a few notable genes, such as *INS*, *PTPN22*, and *CTLA4*, make moderate contributions to risk to T1D [29,37]. For the remaining loci identified to date, though each on its own contributes modestly to T1D, the aggregate seems to increase risk based on PRS studies. The methods of generation of T1D PRS have evolved from the use of GWAS and historical immunological and histocompatibility studies. Initial T1D PRS studies that used four SNVs in the HLA region and seven non-HLA SNVs yielded an AUC-ROC of 0.81. Subsequent studies have yielded an AUC-ROC of 0.84. PRS studies using ancestry-specific variants from non-European cohorts very much perform well, with a reported AUC-ROC of 0.871 in an African cohort. Current studies suggest that the usefulness of a PRS in identifying T1D cases has an AUC-ROC in the range of 0.87–0.93 and can be used to improve screening. The methodology used in a PPRS does not seem to lend itself to easy applicability to T1D due to heterogeneity in pathogenesis. The final common pathway to T1D development is onset of pancreatic islet autoimmunity, and there is a need to identify the relevant biomarkers that can be grouped to allow for PPRS use in predicting risk.

## 3. Type 2 Diabetes (T2D)

Type 2 diabetes results from insulin resistance and insufficient insulin production. T2D accounts for about 90% of all cases of diabetes mellitus. T2D prevalence is increased with obesity, consumption of an energy-dense diet rich in sugar and fat, sedentary lifestyle, stress, and aging. The increasing incidence of T2D is mainly driven by the obesity epidemic [4,5]. T2D is a heterogeneous polygenic disorder with environmental modifiers. In genetically susceptible individuals, there is a slow progression from euglycemia to hyperglycemia, due to a combination of insulin resistance and defects in insulin secretion. Initially, insulin production increases to offset insulin resistance, but eventually there is relative insulin deficiency. The progressive pathogenesis of T2D depends on an interaction between the genetic and environmental factors involved in both the initiation and progression of the disease. Phenotypic features include chronic hyperglycemia, relative insulin deficiency, and insulin resistance (associated with obesity and acanthosis nigricans). The complications of chronic hyperglycemia include nephropathy, neuropathy, retinopathy, and cardiovascular disease [5]. Hyperglycemia in T2D can be controlled by diet and insulin sensitizers and insulin secretagogues, but exogenous insulin may be required as beta cell insufficiency develops. The heritability of T2D is estimated to be between 25% and 70%, and the evidence of genetics contribution comes from studies that have showed high prevalence in certain racial groups, familial aggregation, familial transmission patterns, higher concordance among MZ twins than in DZ twins, and a high sibling risk ratio of 3.5 [1,7,13,14,22]. Early reports suggested that racial differences with Pima Indians (Akimel O’odham) had a higher incidence than other racial groups, but further studies comparing ancestral groups in Mexico showed that lifestyle changes associated with Westernization play a major role in the global epidemic of T2D [38,39].

Several gene loci associated with insulin resistance and defects in insulin secretion have been identified [1,7,40]. Many of these genes identified to date confer modest risk to T2D and yield inconsistent results in replication studies. For instance, the initial candidate genes, peroxisome-proliferation activated receptor gamma (*PPARG*), Calpain 10 (*CAPN10*), and pancreatic beta-cell inwardly rectifying potassium channel Kir 6.2 (*KCNJ11*), identified among many reported association studies failed to replicate in other studies. Non-coding variants in or near the transcription factor 2, hepatic (*TCF2* also known as *HNF1B*), and Wolfram syndrome 1 (*WFS1*) genes have shown strong association with T2D. Severe pathogenic variants in these genes are known to cause rare genetic syndromes that have diabetes as associated findings [1,41,42]. The gene locus transcription factor 7-like-2 gene (*TCF7L2*) has, however, been consistently replicated in diverse populations in Europe, Africa, and Asia, and is considered the strongest common genetic association with T2D in most ethnicities [43,44,45,46,47,48]. Common variants identified by progressive GWAS reportedly explain 20% of the heritability in T2D, suggesting there is “missing heritability” that must be accounted for the low heritability estimate. This may be explained in part by rare variants that are not detected by current analytic methods, sample size considerations, or intronic or intergenic variants [49,50]. Other factors predisposing to T2D may be gene–gene interactions, epigenetic factors related to intrauterine environment, diet, exercise and other lifestyle exposures, copy number variations, noncoding RNAs that affect gene–environment interactions, and gene–environment interactions [1,40]. The establishment of global consortia and biobanks has catalyzed the performance of large-scale genomic studies that have resulted in the identification of thousands of loci associated with T2D. Next-generation sequencing may identify rare variants with large effects, but these may be ancestry-specific [49,50]. The genetic architecture for T2D is markedly more polygenic than T1D, and a T2D PRS does not seem to perform as well. More than 10-fold T2D variants have been reported than for T1D, and none of these variants have the effect sizes analogous to those of T1D in contributing to disease risk. For instance, the common TCF7L2 locus for T2D in the homozygous state confers a 1.7-fold disease risk compared to the 37-fold risk conferred by the DR3-DQ2/DR4-DQ8. More so, an application of the PRS is limited to additive statistical modeling. A multivariate method that combined genetic variants and clinical variables in European populations at best yielded an AUC-ROC in the 0.66 to 0.72 range. The inclusion of common and rare genetic modifiers in multi-ancestry populations improved the AUC-ROC to 0.75. It appears the PPRS may perform better with T2D. Table 2 shows the PRS performance among different populations for T1D and T2D. For instance, the Chinese cohort PRS model discriminates T1D better than T2D when compared to European cohorts [51]. In addition, the African ancestry cohort PRS model discriminates T1D better when the model is compared to European ancestry [31]. For T1D, European ancestry models have better prediction when compared to multi-ancestry model.

## 4. Monogenic Diabetes Mellitus

Monogenic diabetes comprises several clinical dysglycemic disorders that include neonatal diabetes, maturity-onset diabetes of the young (MODY), and several rare genetic syndromes that have diabetes either as an associated finding and/or as a complication [32,33]. Monogenic diabetes as an entity is rare, and may account for 1.5% to 2% of all cases of diabetes [5], but it is believed to be higher as it may be misdiagnosed as T1D or T2D [52]. Monogenic diabetes gene(s) may have incomplete penetrance and variable expressivity leading to different ages of onset and variable presentation even in the same family. These may have led to some patients labelled as having T2D. Monogenic diabetes is mostly caused by impaired development or function of pancreatic beta cells resulting in defective insulin secretion in the absence of obesity. Most patients with MODY or neonatal diabetes have autosomal dominant inheritance. Autosomal and X-linked recessive inheritance account for the remainder. With the advent of next-generation sequencing, several subtypes of monogenic diabetes have been identified with many of the pathogenic variants identified in the *GCK* and *HNF1A* genes [32].

***(i)***  ***Maturity-onset diabetes of the young (MODY)***

MODY is the most common form of monogenic diabetes and may account for 0.5% to 5% of all non-autoimmune diabetes based on predominantly childhood diabetes registries of European cohorts [1,32]. Although MODY has been typically described in Europeans, it has been reported in other racial groups [1,32]. MODY was first described as a mild and asymptomatic form of diabetes that was observed in non-obese children, adolescents, and young adults with improvement in blood glucose levels with sulfonylureas therapy [53]. A clinical diagnosis of MODY should be suspected in an individual with early onset diabetes in adolescence or young adult typically under 35 years of age, strong family history suggesting autosomal dominant inheritance, retained C-peptide greater than 0.6 ng/mL, features atypical for T1D or T2D, mild but stable fasting hyperglycemia that does not progress or respond appreciably to drug therapy, extreme sensitivity to sulfonylureas, personal or family history of neonatal diabetes or neonatal hypoglycemia, and extra-pancreatic features [1]. Table 3 shows the discriminant features between MODY, T1D, and T2D. There are pathogenic variants in at least 14 genes that cause MODY; these include *GCK*, *HNF1A*, *HNF4A*, *HNF1B*, *INS*, *NEUR01*, *PDX1*, *PAX4*, *ABCC8*, *KCNJ11*, *CEL*, *BLK*, and *APPL1*. The four most common genes that cause MODY are *GCK* (MODY2), *HNF1A* (MODY3), *HNF4A* (MODY1), and *HNF1B* (MODY5). MODY is generally inherited in an autosomal dominant fashion but de novo variants do occur. Biallelic pathogenic variants in *GCK* and *PDX1* cause permanent neonatal diabetes mellitus (PNDM) [54,55,56,57].

***(ii)***  ***Neonatal diabetes mellitus (NDM)***

Neonatal diabetes mellitus (NDM) is relatively rare with a prevalence of 1 in 95,000 to 1 in 400,000 [54]. About 50% to 60% of NDM cases have transient hyperglycemia and 40–50% have persistent hyperglycemia [58,59,60]. NDM has been reported in all ethnic groups and there is no gender predilection. This group of disorders are caused by gene variants that result in glucose intolerance with or without pancreatic degeneration [1,58,59,60].

Transient neonatal diabetes mellitus (TNDM) is genetically heterogeneous. Hyperglycemia presents in the neonatal period with remission during infancy but can reoccur during the teenage years. Treatment often starts with intravenous insulin to correct hyperglycemia and dehydration, and long-term treatment with subcutaneous insulin injection or insulin pump. Approximately 50% of cases do not require insulin and are treated with sulfonylurea. Clinical manifestations include severe intrauterine growth restriction, hyperglycemia beginning in the first few weeks of life, dehydration, congenital anomalies, and some dysmorphic features, such as facial dysmorphism, macroglossia, umbilical hernia, deafness, and neurologic dysfunction [60]. Ketoacidosis is rare in TNDM. About 20% of patients have developmental delay and seizures [56]. Heterozygous pathogenic variants in the *KCNJ11* and *ABCC8* genes on 11p15.1 and alterations in 6q24 chromosomal region either due to partial or complete paternal uniparental disomy (UPD6), partial duplication of paternal origin, maternal imprinting defects (maternal hypomethylation), or biallelic *ZFP57* pathogenic variants cause TNDM [60]. In 6q24-transient neonatal diabetes mellitus, there is overexpression of the pleomorphic adenoma gene-like1 (*PLAGL1/ZAC*) and hydatidiform mole-associated and imprinted transcript (*HYMA1*) genes that result in functional beta cell defects [60]. Diabetes mellitus 6q24-TNDM caused by paternal UPD6 is typically de novo; paternal 6q24 duplication could be a de novo event, inherited from a father in a dominant fashion or inherited as a complex chromosomal rearrangement.

Permanent neonatal diabetes mellitus (PNDM) has onset in the first 6 months of life and insulin deficiency is partial or complete. Clinical presentation includes hyperglycemia, ketosis, glycosuria, ketonuria, polyuria, severe dehydration, and history of intrauterine growth restriction. Pathogenic variants in the *ABCC8* and *INS* genes inherited in an autosomal dominant or autosomal recessive fashion cause some cases of PNDM; heterozygous pathogenic variants in *GATA6*, *HNF1B*, and *KCNJ11* that cause autosomal dominant PNDM and *EIF2AK3*, *GCK*, *GLIS3*, *MNX1*, *NEUROD1*,* NKX2-2*, *PDX1*, *PTF1A*, *RFX6*, *SLC2A2*, and *SLC19A2* are autosomal recessive genes that cause PNDM. Consideration should be given to infants with PNDM and extra-pancreatic features. These syndromic disorders are inherited in autosomal dominant, autosomal recessive, or X-linked recessive fashion and gene panels or genomic sequencing will help unravel the diagnosis. Most individuals with autosomal dominant PNDM caused by heterozygous pathogenic variants in the *ABCC8*, *INS*, and *KCNJ11* are de novo [58,59,60,61].

## 5. Gestational Diabetes

Gestational diabetes mellitus (GDM) presents as newly developing hyperglycemia in pregnant women with no previous history of diabetes. GDM affects 9% of pregnancies worldwide and is typically diagnosed at 24–28 weeks of pregnancy. GDM is increasing globally due to obesity, an older maternal age, and a sedentary lifestyle [62,63]. The pathogenesis of GDM is explained by the failure of pancreatic beta cells to produce enough insulin to meet the metabolic demands of pregnancy. During pregnancy, insulin resistance develops in response to placental hormones which leads to an increased production of insulin by pancreatic beta cells. The hyperinsulinemia of pregnancy plays a vital role in promoting glucose uptake by skeletal muscle and adipose tissue and in suppressing glucose production by the liver. Hyperinsulinemia also promotes lipogenesis and increases energy storage in adipose tissue. GDM occurs when maternal beta cells cannot adapt to the metabolic requirements of the mother and fetus associated with pregnancy. GDM is associated with a higher risk of fetal macrosomia, premature birth, hypoglycemia at birth, shoulder dystocia, and difficult delivery due to shoulder dystocia. The infant of a mother with GDM also has a higher risk of obesity, T2D, and CVD [62]. This will suggest genetic susceptibility with environmental triggers as in T2D, and several genes, like the *TCF7L2*, *GCK*, and *IRS1*, have been implicated.

GDM is a heterogeneous disorder; the diabetes resolves in some patients while other patients progress to T2D [62]. Some patients diagnosed with GDM may likely have T1D based on evidence that there is an increased prevalence of the HLA-DR3/DR4 antigens among these women compared to racially matched pregnant women without diabetes [63]. In addition, the evidence suggests that approximately one third of these women with GDM have anti-islet cell antibodies [64].

## 6. Genetic Syndromes Associated with Diabetes Mellitus

Numerous syndromes are associated with diabetes mellitus, and the genetic etiologies are well documented [1,65,66]; these include single gene disorders, chromosomal aberrations, and some triple repeat expansion disorders. Common clinical manifestations of syndromic diabetes include seizures, sensorineural hearing loss, ataxia, vision loss, and developmental abnormalities. Some of the known chromosome disorders associated with diabetes mellitus include Down syndrome, Klinefelter syndrome, Turner syndrome, and Prader–Willi syndrome. Some of the single gene disorders associated with diabetes mellitus, though individually rare, are numerous; some of these are listed in Table 4. Notable disorders include Wolfram syndrome (DIDMOAD—diabetes insipidus, insulin-deficient diabetes mellitus, optic nerve atrophy, and deafness), IPEX syndrome, Rabson–Mendenhall syndrome, Alstrom syndrome, and Wolcott–Rallison syndrome, among others. Repeat expansion disorders, such as Friedreich’s ataxia, Huntington disease, and myotonic dystrophy, do have high rates of diabetes mellitus. Glycogen storage disease type 1, acute intermittent porphyria, cystic fibrosis, and hemochromatosis have increased rates of diabetes mellitus [1,8,66].

The development of affordable high-throughput next generation sequencing techniques using exome and genome sequencing platforms, development of powerful statistical tools, and computational software will help identify the genes involved in many rare syndromes that have diabetes as associated findings and, by doing so, study the common variants in these genes that may help identify the loci involved in diabetes [67].

## 7. Mitochondrial Disorders and Diabetes

Mitochondria are cytoplasmic organelles that produce the energy source ATP for most chemical reactions in the body. They contain their own distinct genome. They are always maternally inherited because mitochondria are transmitted by the ova not the sperm. There are multiple copies (up to thousands) of mtDNA in each cell. Primary mitochondrial disorders arise because of mitochondrial respiratory chain dysfunction, and they affect the tissues and organs that are highly dependent on aerobic respiration [68]. Mitochondrial disorders affect approximately 1 in 5000 individuals [69]. Some mitochondrial disorders affect only a single organ, such as the eye in Leber hereditary optic atrophy and the ear in non-syndromic hearing loss with or without aminoglycoside sensitivity, but many involve multiple organ systems. They are a heterogeneous group of disorders that are caused by pathogenic variants in genes encoding the mitochondrial respiratory chain and related proteins. Mitochondrial disorders that involve nuclear genes are inherited in a Mendelian fashion (nDNA), whereas maternally inherited disorders are inherited strictly from the maternal line (mtDNA). Maternal line DNA-related disorders are clinically heterogeneous because of mitochondrial heteroplasmy, and/or modifying genes and environmental influences. Individuals with mitochondrial disorders may present with symptoms involving several organ systems, in particular, organs that require a lot of energy to function. Mitochondrial DNA deletions generally occur de novo and therefore cause disease in one family member with no significant risk to the maternal line. Single point pathogenic variants or duplications may be transmitted down the maternal line. The father of the proband is not at risk of having the disease-causing mtDNA variant, but the mother has the variant and may or may not have symptoms. A male does not transmit the mtDNA variant to his offspring but a female carrying a heteroplasmic mtDNA variant may transmit a variable amount of the pathogenic mtDNA variant to her offspring. The proportion of mtDNA pathogenic variants must exceed a critical threshold level before a cell expresses a biochemical abnormality of the mitochondrial respiratory chain (a phenomenon called the threshold effect). The percentage level of mtDNA pathogenic variants may vary among individuals within the same family, and among organs and tissues within an individual. The heteroplasmic nature explains the varied clinical phenotype and difficulties with genetic counseling. Common clinical symptoms of mtDNA disorders include neurologic dysfunction, myopathy and/or cardiomyopathy, hearing loss, gastrointestinal disorders, renal tubular dysfunction, hematological disorders, ophthalmological abnormalities, and T2D and pancreatic beta-cell dysfunction [70].

T2D in mitochondrial disorders may progress rapidly to require insulin therapy. Some patients develop GAD and islet cell antibodies creating confusion with late-onset T1D [1,8]. Mitochondrial disorders associated with diabetes mellitus include Kearns–Sayres syndrome, mitochondrial encephalopathy, lactic acidosis, and stroke-like episodes (MELAS), maternally inherited diabetes and deafness (MIDD), and Pearson syndrome [70,71,72]. Affected individuals with these mitochondrial disorders have other organ systems involved in addition to diabetes. Diagnostic approaches involve biochemical, neuroimaging, neurophysiologic, and molecular genetic testing through next generation sequencing techniques.

MELAS is caused by pathogenic variants in the maternally inherited t-RNA gene, and a common pathogenic variant is A3243G. Affected individuals may present with stroke-like episodes, encephalopathy with seizures, myopathy, hearing loss, peripheral neuropathy, short stature, lactic acidosis, and the finding of ragged red fibers on muscle biopsy. Signs and symptoms occur between the ages of 2 and 40 years. Patients may die young due to the severity of the neurologic dysfunction [70,71,72].

Maternally inherited diabetes and deafness (MIDD) is the most common mitochondrial disorder without major neurologic dysfunction of myopathy. It is caused by pathogenic variants in the mitochondrial tRNA; the A3243G variant is commonest. Other variants described include the A8296G and T3721C variants and a 10.4 Kb deletion [70,71].

Kearns–Sayres syndrome is a progressive multisystem disorder that begins before the age of 20 years. Patients present with chronic progressive ophthalmoplegia, pigmentary retinopathy, and cardiac conduction defects. Neurologic dysfunction, hearing loss, endocrinopathies, growth failure, and other body systems dysfunction occur. The disorder is sporadic and is caused by a single large-scale mitochondrial tRNA deletion [72].

Pearson syndrome is usually sporadic, and is the result of a deletion in the tRNA gene. Individuals present with sideroblastic anemia, pancytopenia, pancreatic exocrine insufficiency, renal tubular defect, growth failure, and early death in most cases [72].

## 8. Metabolic Disorders with Erroneous Diabetes Mellitus Diagnosis

Finally, some inborn errors of metabolism (metabolic disorders) may erroneously be diagnosed as diabetes mellitus. Some of these disorders present with severe ketoacidosis and hyperglycemia during metabolic decompensation. Hyperammonemia may be present in some cases. Some of these disorders include the organic acidemias–propionic, methylmalonic, isovaleric acidemias, beta-ketothiolase deficiency (3-oxo-thiolase deficiency), 3-methylglutaconic aciduria, and congenital disorders of glycosylation (CDG). However, CDG typically does not have the ketoacidosis hallmark of the organic acidemias. Recurrent pancreatitis in maple syrup urine disease can result in hyperglycemia [73,74,75].

Comprehensive biochemical genetics work-ups and metabolic gene panel testing will help with diagnosis and appropriate management.

## 9. Conclusions

Diabetes mellitus is a common condition seen in all populations. Family studies have shown that there is genetic susceptibility to T1D and T2D, but the causative genes have eluded researchers. GWAS have identified several gene loci that increase susceptibility to T1D and T2D. Use of PRS and PPRS have enabled risk assessment in susceptible families. Monogenic diabetes mellitus is inherited in a Mendelian fashion, and includes neonatal diabetes, several types of MODY, trinucleotide repeat expansion disorders, mitochondrial cytopathies, and rare genetic syndromes with diabetes as associated findings. Diabetes mellitus is seen in some chromosomal syndromes. Some metabolic disorders have biochemical findings that cause an erroneous diagnosis of diabetes. Clearer definition of the diabetes phenotype, development of powerful statistical methodologies, use of next-generation sequencing applications to interrogate the genome, incorporation of epigenetic mechanisms, and accurate curation of gene variants will help us realize the application of genomic medicine and inform diabetes care.

## Figures and Tables

**Table 1:** Diagnostic criteria for diabetes.

	Diabetes	Impaired Glucose Tolerance
**Fasting plasma glucose level **	≥126 mg/dL	100–125 mg/dL
**2-h plasma glucose level after oral glucose tolerance test (OGTT)**	≥200 mg/dL	140–199 mg/dL
**HbA1c**	≥6.5%	5.7–6.4%
**Random plasma glucose level in the presence of symptoms**	≥200 mg/dL	

**Table 2:** PRS performance (AUC-ROC) across populations for T1D and T2D.

T1D AUC-ROC Values	
European ancestry cohort	0.866–0.923
Multi-ancestry cohort	0.828–0.892
European cohort versus African ancestry cohort [31]	0.798, 0.871
AUC-ROC for T1D versus T2D [51]	
Chinese cohort versus European cohort	0.869, 0.793

**Table 3:** Discriminating MODY from type 1 and type 2 diabetes.

	MODY	T1D	T2D
**Age of onset **	Usually <25–30 years	Young (peak 4–7; 10–14 years) or adult	Adult (usually 45 years or older; younger age in individuals with overweight or obesity
**Autoantibody**	Negative	Positive	Negative or positive
**C-peptide**	>0.6 ng/mL with hyperglycemia	Low to undetectable	High
**HbA1c**	Mild–moderate	High	High
**Ketoacidosis**	Very rare	Severe if there is no insulin treatment	Rare
**BMI**	Lower	Lower	Usually high
**Family history**	Autosomal dominant pattern	Polygenic	Polygenic
**HLA status**	Negative	HLA-DR3 and DR4	Negative
**Insulin sensitivity**	Usually Normal	Usually Normal	Insulin resistance
**Treatment**	Varies by gene. Some respond well to sulfonylureas	Insulin	Insulin sensitizers; insulin secretagogues; insulin

**Table 4:** Selected genetic syndromes associated with diabetes mellitus.

Syndromes	Types of DM	Pattern of Inheritance	Genes
Wolfram syndrome (DIDMOAD)	Type 1	AR	WFS1
Alstrom syndrome	IGT–Type 2	AR	ALMS1
Wolcott–Rallison syndrome	Type 1	AR	EIF2AK3
Bardet–Biedl syndrome	IGT–Type 2	AR (could be oligogenic)	Several genes (at least 26)
Berardinelli–Seip congenital lipodystrophy	IR–Type 2	AR/AD	AGPAT2 BSCL2
Woodhouse–Sakati syndrome	Type 2	AR	DCAF17
H syndrome	Type 1	AR	SLC29A3
Primrose syndrome	IGT–Type 2	AD (de novo in all cases)	ZBTB20
Schmidt syndrome	Type 1	AR/AD/polygenic	
Johanson–Blizzard syndrome	Type 1	AR	UBR1
Laron dwarfism II	Type 2	AR	GHR
Hereditary pancreatitis	IGT–Type 1	AD	PRSS1, SPINK1, CFTR
Ataxia telangiectasia	Type 2	AR	ATM
Stiff Person syndrome	Type 1	AD/Multifactorial (mostly sporadic)	Unknown
Cockayne syndrome	IGT	AR	ERCC6, ERCC8
Werner syndrome	Type 2	AR	RECQL2
IPEX syndrome	Type 1 (Congenital)	XR	FOXP3
Leprechaunism	IR	AR	INSR
Rabson–Mendenhall syndrome	IR	AR	INSR
Bloom syndrome	Type 2	AR	RECQL3/BLM
Mulvihill–Smith syndrome	Type 1	AR	Unknown
Roussy–Levy syndrome	Type 2	AD	PMP22, MPZ
Ramon syndrome	Type 1	AR	Unknown
Prader–Willi syndrome	Type 2	15q abnormality (deletion, UPD, imprinting defect	Contiguous gene deletion, imprinting defect including SNRPN
Hereditary panhypopituitarism	Type 2	AR/XR	PROP1, SOX3
Congenital malabsorptive diarrhea type 4	Type 1	AR	NEUROG3

DM, diabetes mellitus; AR, autosomal recessive; AD, autosomal dominant; XR, X-linked recessive; IGT, impaired glucose tolerance; IR, insulin resistant; UPD, uniparental disomy.

## Data Availability

No new data were created or analyzed in this study.
